# Silica-Gentamicin Nanohybrids: Synthesis and Antimicrobial Action

**DOI:** 10.3390/ma9030170

**Published:** 2016-03-05

**Authors:** Dina Ahmed Mosselhy, Yanling Ge, Michael Gasik, Katrina Nordström, Olli Natri, Simo-Pekka Hannula

**Affiliations:** 1 Department of Materials Science and Engineering, School of Chemical Technology, Aalto University, 02150 Espoo, Finland; yanling.ge@aalto.fi (Y.G.); michael.gasik@aalto.fi (M.G.); simo-pekka.hannula@aalto.fi (S.-P.H.); 2 Microbiological Unit, Fish Diseases Department, Animal Health Research Institute, Dokki, 12618 Giza, Egypt; 3 Department of Biotechnology and Chemical Technology, School of Chemical Technology, Aalto University, 02150 Espoo, Finland; katrina.nordstrom@aalto.fi (K.N.); olli.natri@aalto.fi (O.N.)

**Keywords:** silica nanoparticles, gentamicin, *in vitro* release, antimicrobial effect, orthopedic applications

## Abstract

Orthopedic applications commonly require the administration of systemic antibiotics. Gentamicin is one of the most commonly used aminoglycosides in the treatment and prophylaxis of infections associated with orthopedic applications, but gentamicin has a short half-life. However, silica nanoparticles (SiO_2_ NPs) can be used as elegant carriers for antibiotics to prolong their release. Our goal is the preparation and characterization of SiO_2_-gentamicin nanohybrids for their potential antimicrobial administration in orthopedic applications. *In vitro* gentamicin release profile from the nanohybrids (gentamicin-conjugated SiO_2_ NPs) prepared by the base-catalyzed precipitation exhibited fast release (21.4%) during the first 24 h and further extension with 43.9% release during the five-day experiment. Antimicrobial studies of the SiO_2_-gentamicin nanohybrids *versus* native SiO_2_ NPs and free gentamicin were performed against *Bacillus subtilis* (*B. subtilis*), *Pseudomonas fluorescens* (*P. fluorescens*) and *Escherichia coli* (*E. coli*). SiO_2_-gentamicin nanohybrids were most effective against *B. subtilis*. SiO_2_ NPs play no antimicrobial role. Parallel antimicrobial studies for the filter-sterilized gentamicin were performed to assess the effect of ultraviolet (UV)-irradiation on gentamicin. In summary, the initial fast gentamicin release fits the need for high concentration of antibiotics after orthopedic surgical interventions. Moreover, the extended release justifies the promising antimicrobial administration of the nanohybrids in bone applications.

## 1. Introduction

Orthopedic applications such as bone implants and open fractures necessitate therapeutic and prophylactic administration of antibiotics [1]. Microorganisms can attach onto the nails used for stabilization of fractured-bones and may lead to systemic antibiotic resistant biofilms [2]. The traditional systemic administration of antibiotics shows poor penetration to the infected tissues [3]. However, delivery systems that are able to locally release the antibiotics can maintain antibiotic concentrations at the infected sites until healing is complete [4], reduce the frequency of drug administration [5,6] and, consequently, enhance patient compliance [7]. Materials implemented as carriers for antibiotics should have release kinetics that comply with the requirements to treat the infection [8] and should be biodegradable to exclude further surgical intervention to remove them [3,9].

Gram-negative bacteria are increasingly associated with the risk of infections leading to osteomyelitis, which has been more traditionally attributed to gram-positive bacteria, especially *Staphylococcus* species. This increasing risk is due to the high number of administration of orthopedic implants. However, there are inadequate data regarding the involvement of gram-negative bacteria in osteomyelitis [10]. Gentamicin is an aminoglycoside that is ideally used in treatment of osteomyelitis. A potent broad-spectrum antibiotic that is effective against gram-positive and gram-negative bacteria [11]. Controlled release of aminoglycosides is desirable, as their half-life is short and their bioavailability is low, which is a challenge for the conventional treatment modalities [12].

In recent decades, much attention has been paid to silica nanoparticles (SiO_2_ NPs) as promising carriers for controlled drug delivery [13,14]. Studies have been performed to investigate the role of SiO_2_ xerogels as carriers to enable the controlled release of drugs. Aughenbaugh *et al.* [8] studied the administration of SiO_2_ NPs prepared using different water/alkoxysilane molar ratios (4, 6 and 10) as carriers for vancomycin and detected that the high molar ratio SiO_2_ gels possessed faster vancomycin release than the low molar ratio ones. Shi *et al.* [15] examined the *in vitro* sustained release of gentamicin from poly(lactide-*co*-glycolide)/mesoporous SiO_2_-hydroxyapatite composite material (PLGA/HMS-HA). The release rate of gentamicin from HMS-HA particles was extended to one month suggesting that PLGA/HMS-HA scaffolds are promising drug delivery materials for orthopedic applications. Xue and Shi [6] suggested also that PLGA/mesoporous SiO_2_ hybrid structure is an ideal drug release material for bone filling applications. Gentamicin exhibited a sharp initial burst for one day followed by a slow release from the loaded SiO_2_ for three weeks. By the encapsulation with PLGA, the release period can be extended to five weeks.

The aforementioned studies have only demonstrated the key role played by SiO_2_ NPs as carriers for antibiotics especially in bone applications. Moreover, none of the previous studies have dealt with the antimicrobial properties of the antibiotic-loaded SiO_2_ NPs that enable their administration in different orthopedic applications. Accordingly, in the present paper, we report the antimicrobial performance of the synthesized SiO_2_-gentamicin nanohybrids. To the best of our knowledge, only a few studies have been implemented regarding the antimicrobial action of gentamicin-conjugated SiO_2_ NPs. Corrêa *et al.* [16] detected that SiO_2_-encapsulated gentamicin prepared by precipitation route and thus having a high amount of gentamicin on the surface of SiO_2_ NPs exhibited effective antimicrobial action against *Staphylococcus aureus* (*S. aureus*). However, the previous work was not coupled with mechanistic studies for the gentamicin release nor for the bacterial interactions with the gentamicin-conjugated SiO_2_ NPs. Agnihotri and coworkers [13] examined the antimicrobial action of aminoglycoside-conjugated SiO_2_ NPs and detected improved antimicrobial properties of the conjugate against kanamycin-resistant *E. coli*. However, as in other prior studies, the aminoglycoside release studies were not run concurrently with the antimicrobial studies. Therefore, the role of SiO_2_ NPs as prime antibiotic carriers that permit the extended release of antibiotics to be implemented in orthopedic applications was not demonstrated. The present study aims to demonstrate the controlled release of gentamicin from the SiO_2_-gentamicin nanohybrids through the *in vitro* release studies. The present work makes an original contribution by performing the antimicrobial studies concurrently with the *in vitro* release studies in order to estimate the amount of gentamicin released from the SiO_2_-gentamicin nanohybrids. Consequently, appropriate concentrations of the nanohybrids that will supply therapeutic levels of the gentamicin can be applied to avoid the possible sub-inhibitory released concentrations of the antibiotic. Furthermore, our work is designed to shed light on the antimicrobial actions facilitated by the SiO_2_-gentamicin nanohybrids in comparison with free gentamicin and native SiO_2_ NPs to exclude any antimicrobial role that could be played by the SiO_2_ NPs and to demonstrate that the antimicrobial effect can be attributed to the antibiotic.

In the present study, we examine the antimicrobial action of the tested materials against different bacterial species and cover the lack of attention that the gram-negative bacteria have received regarding their role in osteomyelitis. This lack of concern was addressed by performing antimicrobial studies against different laboratory strains of *E. coli* and *P. fluorescens* as models of the gram-negative bacteria that are reported in causing osteomyelitis. As our study does not focus on the clinical etiology of osteomyelitis, nor are we looking to identify causative agents of this condition, we selected *B. subtilis* as a model organism for gram-positive bacteria, as studies with *S. aureus* would require working in Biosafety level-II facilities. Moreover, as the present study primarily focuses on shedding insights on differences between the antimicrobial sensitivities of gram-negative and gram-positive bacteria in a setting where the antibiotic is delivered in the presence of the SiO_2_-gentamicin nanohybrids, *B. subtilis* was chosen as a representative of gram-positive bacteria. Moreover, in this paper we strive to complement previously published work by evaluating the effect of sterilization on the loaded-gentamicin before the antimicrobial application through performing parallel antimicrobial studies for the filter-sterilized gentamicin in comparison to the UV-irradiated gentamicin.

The conventional high processing temperatures of SiO_2_ NPs may affect the organic molecules, but this can be overcome as sol-gel technology can be accomplished at room temperature [5]. Therefore, this work is targeted towards the preparation of the SiO_2_-gentamicin nanohybrids and native SiO_2_ NPs at ambient temperature through a single step sol-gel procedure. The present study focuses on the antimicrobial action of the SiO_2_-gentamicin nanohybrids against gram-positive and gram-negative bacteria. The specific focus is on the elucidation of the possible use of the SiO_2_-gentamicin nanohybrids in the treatment and prophylaxis of bone infections and orthopedic applications. Moreover, Transmission electron microscopy (TEM) studies were performed to detect the antibacterial mechanistic action of the SiO_2_-gentamicin nanohybrids on the microscopic level. The interaction of the native SiO_2_ NPs and free gentamicin with the bacterial cells was also studied. The main objective of this study is preparation of SiO_2_-gentamicin nanohybrids to demonstrate that the released gentamicin has the extended antimicrobial action and could therefore be used efficiently for targeting infectious microorganisms.

## 2. Results and Discussion

### 2.1. Characterization of the SiO_2_-Gentamicin Nanohybrids and Native SiO_2_ NPs

The SiO_2_-gentamicin nanohybrids prepared by the base-catalyzed precipitation route were characterized by using different series of instrumental characteristic techniques to determine the different properties of the SiO_2_-gentamicin nanohybrids and native SiO_2_ NPs. By X-ray diffraction (XRD), the native SiO_2_ NPs and SiO_2_-gentamicin nanohybrids showed nearly the same XRD pattern of the broad peak with Bragg angle at 2θ around 24° (Supplementary Figure S1), which indicates that the SiO_2_ NPs used in the present study were amorphous in nature. This amorphous pattern has been reported in previous studies [17].

**Figure 1:**
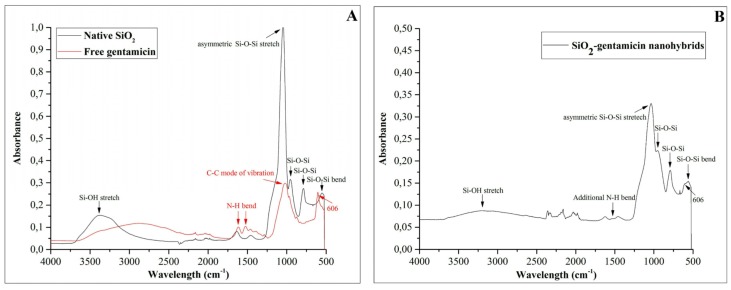
FTIR spectra of the: (**A**) native SiO_2_ NPs and free gentamicin sulfate; and (**B**) SiO_2_-gentamicin nanohybrids.

The Fourier transform infrared (FTIR) spectra of the native SiO_2_ NPs, free gentamicin and SiO_2_-gentamicin nanohybrids are depicted in Figure 1A,B. The native SiO_2_ NPs (Figure 1A) showed Si–O–Si bending at 553, 790 and 955 cm^−1^. In addition, a sharp peak was detected at 1053 cm^−1^ for the asymmetric Si–O–Si stretch and a broad band at 3381 cm^−1^ associated with the Si–OH stretching. Similar spectrum was reported previously [18] for the well polymerized SiO_2_ network. The free gentamicin (Figure 1A) showed a band at 606 cm^−1^ that is considered a major band for gentamicin [19]. Two more bands at 1524 and 1614 cm^−1^ were also detected. These bands can be ascribed to the N–H vibrational bending of primary aromatic amines [20]. The spectrum of SiO_2_-gentamicin nanohybrids (Figure 1B) showed peaks at 559, 793, and 957 and a broad peak at 3199 cm^−1^. This spectrum did not impose significant shifts from the bands of the native SiO_2_ NPs, which can be attributed to the amorphous nature of the SiO_2_ NPs. The intensity of the bands decreased after the conjugation. The present measurements showed in addition a shoulder at 606 cm^−1^ and a band at 1529 cm^−1^ that are obviously originating from the gentamicin. The current results indicate that gentamicin has been loaded to the SiO_2_ NPs and causes no significant changes in the silica xerogel network.

**Figure 2:**
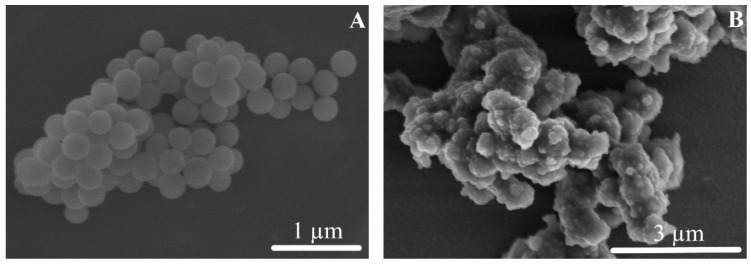
SEM images of the: (**A**) native SiO_2_ NPs with the smooth spherical surfaces; and (**B**) SiO_2_-gentamicin nanohybrids showing the surface roughness that is caused by the loaded gentamicin.

The scanning electron microscopy (SEM) images provided important information for the native SiO_2_ NPs and the SiO_2_-gentamicin nanohybrids as shown in Figure 2. The native SiO_2_ NPs (Figure 2A) presented smooth spherical surfaces. However, the SiO_2_-gentamicin nanohybrids (Figure 2B) showed surface roughness due to the conjugation of gentamicin to the SiO_2_ NPs and at the same time, some nanohybrids are coalesced into larger agglomerates. Our results showed the loading of gentamicin to the SiO_2_ NPs. The detected roughness of the SiO_2_-gentamicin nanohybrids is important, since surface roughness plays a major role in controlling the initial release of antibiotics, as rough surfaces establish a larger area for antibiotic release [21]. Our results suggest that the initial antibiotic release is mainly a surface phenomenon.

**Figure 3:**
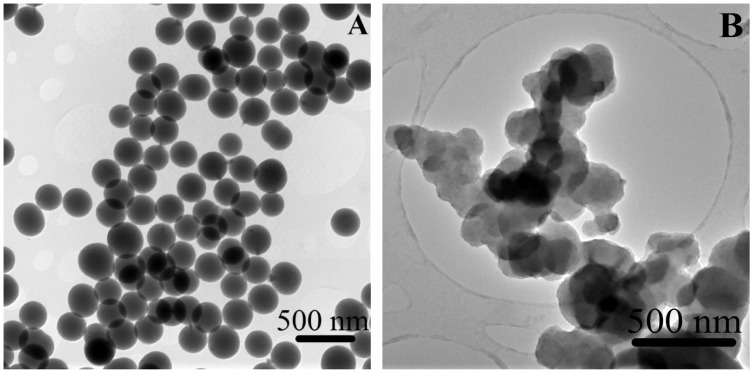

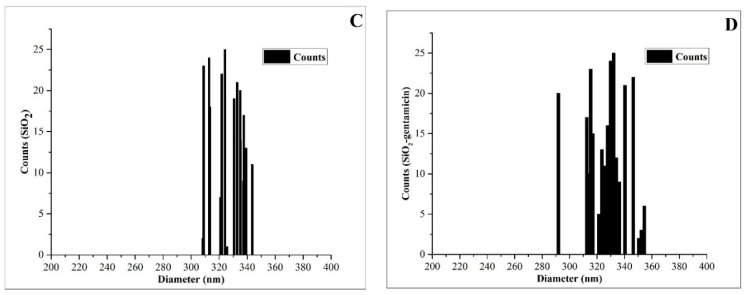
TEM images of the: (**A**) native SiO_2_ NPs showing a homogenous spherical morphology; and (**B**) SiO_2_-gentamicin nanohybrids displaying the conjugated SiO_2_ network in conglomerates. Size distributions of the: (**C**) native SiO_2_ NPs; and (**D**) SiO_2_-gentamicin nanohybrids. The size distribution was determined by measuring the area of each single NP, using the ImageJ software and calculating the average diameter of the measured 25 NPs.

As depicted in Figure 3, the transmission electron microscopy (TEM) image of the native SiO_2_ NPs (Figure 3A) showed a homogenous spherical morphology and an average size of 327 ± 10 nm, with a size range (Figure 3C) of 308 to 339 nm. The SiO_2_-gentamicin nanohybrids (Figure 3B) showed network of conglomerated spherical SiO_2_ NPs of 332 ± 15 nm average size and a size distribution (Figure 3D) of 292 to 354 nm. The slight increase in the size of SiO_2_-gentamicin nanohybrids over that of the native SiO_2_ NPs can be related to the loading of the antibiotic to the SiO_2_ network.

**Figure 4:**
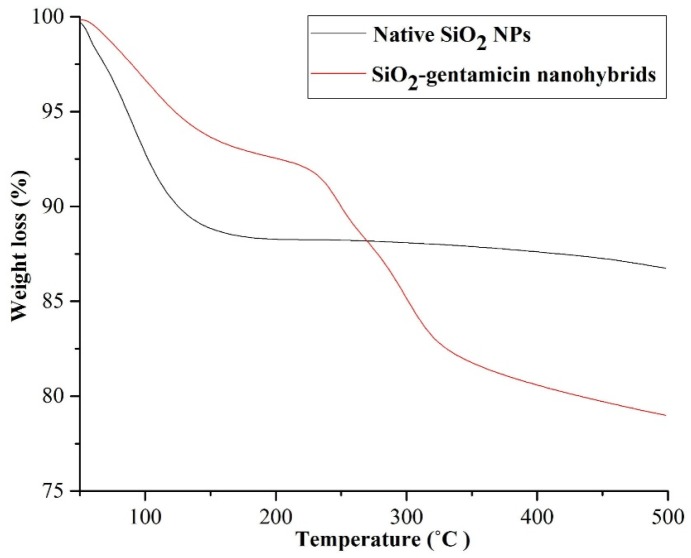
TGA of the native SiO_2_ NPs and SiO_2_-gentamicin nanohybrids. The mass loss from 215 to 500 °C in the SiO_2_-gentamicin nanohybrids was ascribed to gentamicin decomposition.

The mass weight of the overnight dried SiO_2_ NPs and SiO_2_-gentamicin nanohybrids was measured and their weight difference can be attributed to the loaded gentamicin sulfate that theoretically comprises 12.7 wt % payload. The thermogravimetric analysis (TGA) results of the native SiO_2_ NPs (not loaded with the antibiotic) and SiO_2_-gentamicin nanohybrids are shown in Figure 4. The data demonstrate a total mass loss of 12.96% and 20.84% upon heating up to 500 °C in an argon atmosphere for the native SiO_2_ NPs and SiO_2_-gentamicin nanohybrids, respectively. An initial weight loss of 6.94% and 3.18% during heating of the native SiO_2_ NPs and SiO_2_-gentamicin nanohybrids up to 100 °C, respectively, can be ascribed to the removal of the absorbed and residual water. Hence, the mass loss of the native SiO_2_ after heating to 500 °C with the subtracted low-temperature moisture removal is ~6%. The SiO_2_-gentamicin nanohybrids showed further weight loss of 4.4% at 215 °C that can be considered as the start of aminoglycoside decomposition. A prior study [22] has recorded the weight loss of amikacin sulfate at the range of 190 to 270 °C. Other work [23] demonstrated that rising the temperature above 150 to 200 °C resulted in the loss of gentamicin. In the present study, the final weight loss of 13.25% in the range of 220 to 500 °C can be related to the removal of glycosidic moieties [13]. The amount of gentamicin in the SiO_2_-gentamicin nanohybrids can be determined by subtracting the mass loss of native SiO_2_ NPs from the mass loss of SiO_2_-gentamicin nanohybrids after excluding the moisture weight loss in both samples and assuming that SiO_2_ absorbs equivalent amount of water. Therefore, gentamicin sulfate, according to the TGA data, constitutes ~11.7 wt % of the SiO_2_-gentamicin nanohybrids. This is very close to our calculated theoretical loaded gentamicin sulfate (12.7 wt %) to the SiO_2_-gentamicin nanohybrids.

### 2.2. The *in Vitro* Release Profile of Gentamicin from the SiO_2_-Gentamicin Nanohybrids

**Figure 5:**
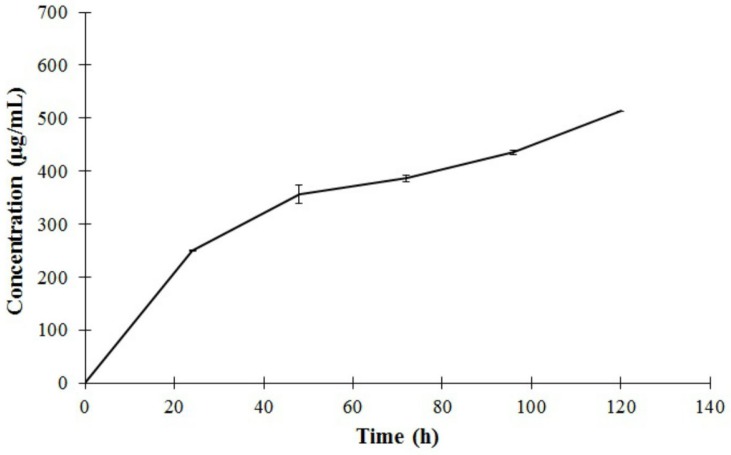
The *in vitro* release of gentamicin from the SiO_2_-gentamicin nanohybrids (10 mg/mL) with a drug concentration of 11.7 wt % in PBS at pH 7.4. The mean cumulative concentration of gentamicin (µg/mL) (each data point is the mean of three measurements) was expressed as a function of immersion time (h). The data were linearly fitted to the gentamicin standards. Error bars represent the standard errors (n= 3).

Another objective of our study, after the detailed characterization of the prepared materials is to detect the sustained gentamicin release from the SiO_2_-gentamicin nanohybrids. The *in vitro* release of gentamicin is depicted in Figure 5. The SiO_2_-gentamicin nanohybrids (10 mg/mL) containing 11.7 wt % gentamicin sulfate demonstrated a cumulative release of 250.5 ± 1.6, 356 ± 29.9, 386 ± 10.3, 435.3 ± 7.8 and 512.8 ± 0.7 µg/mL gentamicin in PBS solution after 24, 48, 72, 96 and 120 h, respectively. This release profile constitutes a fractional release around 21.4%, 30.5%, 33%, 37.2% and 43.9% of the total amount of the loaded-gentamicin sulfate, correspondingly, over the 5 days of the experiment.

The relatively faster gentamicin release during the first 24 h (21.4%) when compared with the slower sustained release during the successive days of the experiment is likely resulting from the initial easily-released gentamicin that is attached to the surface of SiO_2_ NPs. Shi *et al.* [15] recorded even higher initial bursts of gentamicin release (60%) from HMS-HA during the first hour. However, slower release was detected within the following 11 h. They proposed that this initial burst release may have also been due to the release of gentamicin molecules that were adsorbed either on the surface of particles or by physical interaction with HMS-HA.

The subsequent decrease in the gentamicin release rate that is observed in our experiment can be attributed to the chemical equilibrium between the gentamicin-silica chemical bonding and the dissolution of water-soluble gentamicin in the aqueous medium [6]. In the same vein, Kortesuo *et al.* [14] showed slower release of the loaded toremifene citrate and dexmedetomidine HCl from the silica gel microspheres after their primary high release due to the depletion of the drugs from the SiO_2_ surface.

**Figure 6:**
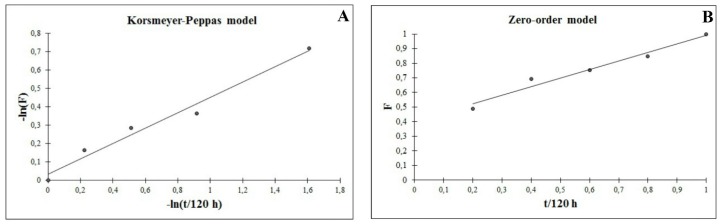
Pharmacokinetic fit of the release profile of gentamicin from the SiO_2_-gentamicin nanohybrids to: (**A**) Korsmeyer–Peppas model; and (**B**) zero-order model. *F* is the drug release fraction at time *t*.

At fitting our release data to various mathematical models, the data (Table 1) showed the best pharmacokinetic fit to Korsmeyer–Peppas model (Figure 6A) with a release rate constant (k)= 0.966, n = 0.416 (n≤0.5 in case of Fickian diffusion) and a good linearity (*R*^2^ value) of 0.9769. This fit suggests that gentamicin release from the SiO_2_-gentamicin nanohybrids is controlled by Fickian diffusion [20,22]. Nevertheless, the release data fit fairly well to first-order model (Figure 6B) with *R*^2^ value of 0.9655; this can explain the initial fast release of gentamicin during the first 24 h. Previous studies have reported that fitting quality is likely better for the initial stage than for the terminal one [24]. We conclude that, gentamicin release is fast at the initial stage, but without an unfavorable initial burst release, followed by slower release during the successive days of the experiment. This release mechanism is due to the amount of gentamicin molecules adsorbed on the surface of the SiO_2_ NPs combined with the gentamicin diffusion from the SiO_2_-gentamicin nanohybrids.

Our findings suggest that the SiO_2_ NPs can be proposed as elegant carriers for gentamicin sulfate that permit the initial fast gentamicin release, which exceeds the minimal gentamicin therapeutic concentration (1 to 4 µg/mL) against different sensitive bacteria [25]. The present findings thus fit the initial need for high concentration of antibiotic after orthopedic surgery and avoid the sub-inhibitory antibiotic concentrations that can provoke a problematic bacterial resistance. In addition, the reported extended release of gentamicin is crucial factor for the efficient implementation of the SiO_2_-gentamicin nanohybrids in versatile orthopedic applications.

### 2.3. Antimicrobial Activity of the SiO_2_-Gentamicin Nanohybrids, SiO_2_ NPs and Free Gentamicin

#### 2.3.1. Agar Diffusion Assay

**Figure 7:**
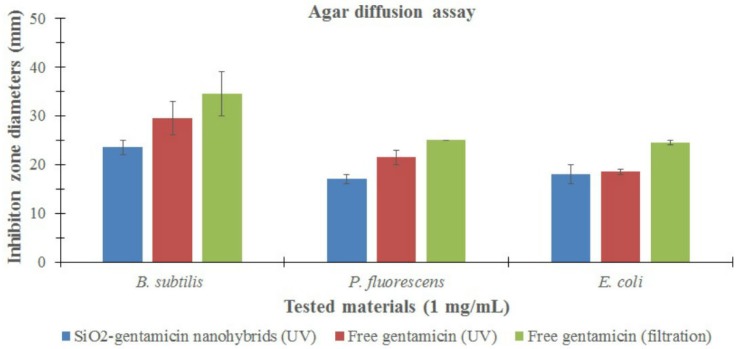
Inhibition zone diameters (mm) of the SiO_2_-gentamicin nanohybrids and free gentamicin (sterilized by UV-irradiation and filtration). Error bars represent the standard errors.

The final objective of our study is to identify the antimicrobial properties of the SiO_2_-gentamicin nanohybrids that could permit their administration in different orthopedic applications. In order to assess this antimicrobial activity, the zones of bacterial growth inhibition were measured for the conjugated and native SiO_2_ NPs in comparison with that of free gentamicin (Figure 7; Supplementary Figure S2). The native SiO_2_ NPs were not antimicrobial, as no zones of inhibition were produced against any of the bacterial species that were tested. Our results indicate that the SiO_2_ NPs have no antimicrobial effect when they are conjugated with antibiotics and act only as carriers for the gentamicin. Therefore, the antimicrobial effect of the SiO_2_-gentamicin nanohybrids is exerted only by the released gentamicin from the SiO_2_-gentamicin nanohybrids.

The agar diffusion results showed that the SiO_2_-gentamicin nanohybrids and free gentamicin have a more marked antimicrobial effect against the gram-positive bacterium, *B. subtilis*, than on the gram-negative bacteria, *P. fluorescens* and *E. coli*. It is evident that the outer membrane of the gram-negative bacteria most probably acts as an extra barrier to the antimicrobial effects of the gentamicin. Gram-positive bacteria lack this outer membrane that may explain their greater sensitivity [16].

#### 2.3.2. MIC (Minimum Inhibitory Concentration) of the SiO_2_-Gentamicin Nanohybrids, Native SiO_2_ NPs and Free Gentamicin

MIC of the tested twofold serially diluted SiO_2_-gentamicin nanohybrids, native SiO_2_ NPs and free gentamicin were determined and are shown in Table 2. The native SiO_2_ NPs did not inhibit the bacterial growth even at the highest concentration (1 mg/mL). These results confirm our negative agar diffusion assay results for the native SiO_2_ NPs and demonstrate that the SiO_2_ NPs have no antimicrobial effect. The MIC of the SiO_2_-gentamicin nanohybrids was 250 µg/mL (that released 6.26 µg/mL gentamicin) against all the bacterial species. When comparing the same tested concentrations of the free gentamicin with that of the SiO_2_-gentamicin nanohybrids, we found that free gentamicin concentration of 0.98 µg/mL inhibited *B. subtilis* and 15.63 µg/mL was effective against *P. fluorescens* and *E. coli*. MIC of the free gentamicin against *P. fluorescens* and *E. coli* is within the range of 3.91 to 15.63 µg/mL that is estimated to be 6.26 µg/mL, which is the amount of released gentamicin from the 250 µg/mL SiO_2_-gentamicin nanohybrids that inhibited the *P. fluorescens* and *E. coli*. Comparing the same concentrations of the tested SiO_2_-gentamicin nanohybrids with that of the free gentamicin is important to prove that the high MIC of the SiO_2_-gentamicin nanohybrids (250 µg/mL) against the bacterial species is governed by the amount of released gentamicin (6.26 µg/mL) and not from the gentamicin itself.

The agar diffusion assay and MIC results show that the antimicrobial efficacy of the SiO_2_-gentamicin nanohybrids is less than that of the free gentamicin. This may be due to the aforementioned release profile of gentamicin from the SiO_2_-gentamicin nanohybrids. After 24 h, the concentrations of 1 mg/mL and 250 µg/mL of the SiO_2_-gentamicin nanohybrids released 25.05 and 6.26 µg/mL gentamicin, respectively. The same concentrations of 1 mg/mL and 250 µg/mL of the free gentamicin are solely gentamicin sulfate, which explains the more potent antimicrobial efficacy of the free gentamicin than that of the SiO_2_-gentamicin nanohybrids. Our data demonstrate that the sustained release of gentamicin from the SiO_2_-gentamicin nanohybrids fits their ideal antimicrobial administration in different orthopedic applications. However, careful considerations should be taken regarding the concentrations of the administered antibiotic-conjugated nanoparticles to avoid the release of sub-inhibitory concentrations of the antibiotic. These sub-inhibitory concentrations may result in the generation of antibiotic bacterial resistance that is elicited mainly from the miss-use of the antibiotic-conjugated nanoparticles that would continuously supply low levels of antibiotic. A previous study [23] has demonstrated a higher resistance of *S. aureus* to gentamicin-conjugated Au NPs (MICs were 220 and 440 μg/mL) than to free gentamicin (MIC was 2 μg/mL) and concluded that this resistance is not elicited from the low efficacy of gentamicin itself, but from the released gentamicin that is interacting with the bacterial cells. Accordingly, we stress that it is important to couple *in vitro* antibiotic release experiments with the antimicrobial assays to exclude any bacterial resistance related to the low concentration of the released antibiotic from the carriers and not from the antibiotic itself. According to our results, 10 mg/mL of the SiO_2_-gentamicin nanohybrids released 250.5 ± 1.6, 356 ± 29.9, 386 ± 10.3, 435.3 ± 7.8 and 512.8 ± 0.7 µg/mL gentamicin after 24, 48, 72, 96 and 120 h, respectively. In this case, the released gentamicin concentration exceeded the MIC of the free gentamicin every 24 h; however, there was a high standard deviation value for the released gentamicin after 48 h. It is therefore evident that attention should be taken in the formulation of these materials for orthopedic applications. Notably, attention should be paid, on the one hand, to the concentrations of the applied materials to avoid the release of sub-inhibitory concentrations of the antibiotic and on the other hand, to avoid possible toxicity, which may result when the aim is to provide adequately high levels of the released antibiotic. More importantly, the parallel study of the antimicrobial properties of the filter-sterilized gentamicin showed non-significant increase in the diameters of the inhibition zones (Figure 7) in comparison to that of the UV-sterilized free gentamicin. The recorded MIC was exactly the same. These experiments show that UV-irradiation can be used as a convenient method for sterilization of the antibiotic-loaded nanoparticles, especially if the size of the loaded nanoparticles is larger than the pore size of the syringe filter (0.2 µm). Similarly, prior literature [26] described that irradiation did not affect the release of gentamicin from the gentamicin-loaded collagen/PLGA microparticle composite.

#### 2.3.3. Bactericidal Mechanistic Action of the SiO_2_-Gentamicin Nanohybrids *versus* Free Gentamicin

**Figure 8:**
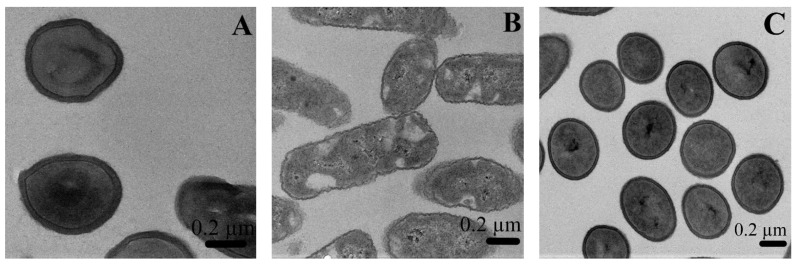

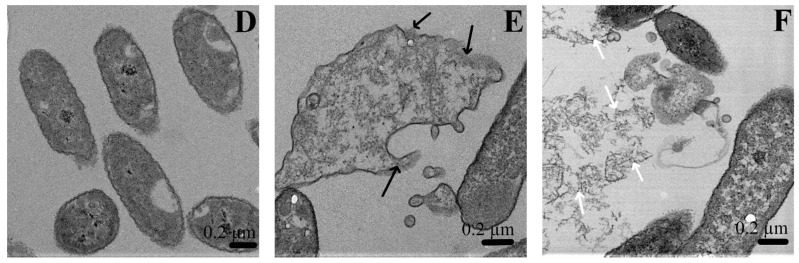
TEM micrographs of the: (**A**) untreated *B. subtilis* and (**B**) *P. fluorescens* (control negative); native SiO_2_ NPs-treated (**C**) *B. subtilis* and (**D**) *P. fluorescens*; and *P. fluorescens* interacted with (**E**) SiO_2_-gentamicin nanohybrids and (**F**) free gentamicin. Black arrows indicate disorganization of the bacterial cell membranes. White arrows show complete deterioration of the bacterial cells.

In order to provide new insights on the antimicrobial properties of the SiO_2_-gentamicin nanohybrids on the microscopic level, TEM studies were performed. The TEM micrographs of the bacterial cells treated with the native SiO_2_ NPs, SiO_2_-gentamicin nanohybrids and free gentamicin, as well as the untreated cells are depicted in Figure 8. The untreated *B. subtilis* (Figure 8A) showed the typical thick cell wall of gram-positive bacteria, while the untreated *P. fluorescens* (Figure 8B) demonstrated the thin cell wall of gram-negative bacteria. The cell wall thickness of gram-positive bacterium *B. subtilis* is about 20–30 nm and formed by interwoven peptidoglycans and secondary polymers. The gram-negative bacteria possess thin peptidoglycan layer with an outer lipopolysaccharide membrane [27].

The native SiO_2_ NPs-treated bacterial cells (Figure 8C,D) showed no morphological changes and the bacterial cells appeared intact and similar to the control untreated cells. These results confirm the lack of any antimicrobial properties for the SiO_2_ NPs at the microscopic level and show that only the gentamicin is responsible for the antimicrobial effect. The SiO_2_-gentamicin nanohybrids-treated bacterial cells (Figure 8E) presented disorganization of the bacterial cell membranes. The free gentamicin-treated bacterial cells (Figure 8F) were more affected than the bacterial cells treated with the SiO_2_-gentamicin nanohybrids. The treatment with the free gentamicin showed complete deterioration of the bacterial cell membranes resulting in the leakage of intracellular contents. The mechanism by which the bactericidal action of the SiO_2_-gentamicin nanohybrids and free gentamicin is exerted can be attributed to the aminoglycoside structure of gentamicin. The binding sites for aminoglycosides are teichoic acids of the cell wall and phospholipids of the cell membrane of gram-positive bacteria [28]. Amongst Enterobacteriaceae members and *Pseudomonas species*, aminoglycosides can cross the cell walls through porin channels and bind to the 30S subunit of ribosome, resulting in errors in protein synthesis and bacterial death [29].

Finally, it should be noted that, our TEM results represent a third method to confirm the agar diffusion assay and MIC outcomes as follows: (1) the SiO_2_ NPs act as carriers for the sustained release of gentamicin and have no antimicrobial effect themselves; and (2) The SiO_2_-gentamicin nanohybrids have a lower antimicrobial effect than the free gentamicin. This correlates with the sustained release profile of gentamicin from the SiO_2_-gentamicin nanohybrids, which supports their potential to be used in different orthopedic applications that require extended antibiotic release. However, it is to be noted that this study has been only focused on model organisms, and not on bacteria isolated from bone infections and associated with osteomyelitis. It would therefore be paramount to use such organisms in further studies to show more concrete evidence for the potential use of SiO_2_-gentamicin nanohybrids for treatment and prophylaxis of osteomyelitis

## 3. Materials and Methods

### 3.1. Synthesis and Characterization of the SiO_2_-Gentamicin Nanohybrids and Native SiO_2_ NPs

The SiO_2_-gentamicin xerogels were prepared via the base-catalyzed precipitation route adopting the method described by Corrêa *et al.* [16]. Briefly, 500 mg of gentamicin sulfate (Sigma-Aldrich, Shanghai, China) was dissolved in 10 mL of tetraethyl orthosilicate (TEOS, Aldrich^®^ chemistry, Steinheim, Germany). Then, 20 mL of ammonium hydroxide (28%–30%, Sigma-Aldrich, St. Louis, MO, USA) was added to the solution. The mixture was stirred for 20 min at room temperature until precipitation. The resultant material was dried overnight at room temperature and then ground. The native SiO_2_ NPs were prepared with the same aforementioned method but excluding the addition of gentamicin sulfate.

Once the materials were prepared, several instrumental techniques were applied for the characterization. The SiO_2_-gentamicin nanohybrids and native SiO_2_ powders were subjected to X-ray diffraction measurements with an X’pert Powder Pro (PANalytical/PW3040/60, Almelo, The Netherlands) operated at generator settings of 40 mA and 45 kV using Cu-Kα radiation and a goniometer scanning (2θ) over 10°–90°. The FT-IR measurements were performed using a Nicolet 380 FT-IR (Thermo Electron Corporation, White Bear Lake, MN, USA) spectrometer in the attenuated total reflection (ATR) mode, with a resolution of 2 cm^−1^ and a scan range of 4000–500 cm^−1^. The spectra were averaged over 64 scans. The samples were analyzed using the absorbance mode. The SEM analyses were accomplished by using a Hitachi SEM (S-4700, Tokyo, Japan) to detect the surface morphology of the synthesized materials. For the SEM studies, the samples of the SiO_2_-gentamicin nanohybrids were coated with a thin carbon film using a cool sputtering device (Leica EM SCD050, Wetzlar, Germany). The TEM studies of the shape and size of the prepared materials were implemented using a Tecnai G2 F20 TEM (FEI, Eindhoven, The Netherlands). The mass changes of the native SiO_2_ NPs and SiO_2_-gentamicin nanohybrids were weighted after overnight drying at room temperature to determine the theoretical concentration of the loaded drug [6,15]. The TGA of the native and conjugated SiO_2_ NPs was performed with a simultaneous thermal and spectral analysis using a STA449C Jupiter (Netzsch Gerätebau GmbH, Selb, Germany) coupled with a Tensor-27 FTIR spectrometer (Bruker Optics, Ettlingen, Germany). The analysis was conducted from 30 to 500 °C at a rate of 10 °C/min following Sharma *et al.* [22], but in an argon atmosphere (flow rate of 20 mL/min). Aluminum crucibles without lids were utilized in the experiment, with an empty crucible as a reference.

### 3.2. The in Vitro Release Studies of Gentamicin from the SiO_2_-Gentamicin Nanohybrids

Gentamicin sulfate absorbs neither ultraviolet nor visible light. Hence, indirect spectrophotometric analysis was performed to detect the concentrations of the antibiotic using derivatization. Phthaldialdehyde reagent (Sigma-Aldrich, St. Louis, MO, USA) acted as a derivatizing agent that interacts with the amino groups of gentamicin sulfate to produce a chromophoric product [30]. The *in vitro* release studies were performed in phosphate-buffered saline solution (PBS, pH 7.4) at 37 °C. The SiO_2_-gentamicin nanohybrids (20 mg) were placed in tubes containing 2 mL PBS (10 mg/mL). The tubes were incubated at 37 °C and shaken at 150 rpm (Kühner incubator shaker, Lab-Therm, Fennolab, Basel, Switzerland) for regular time intervals of 24, 48, 72, 96 and 120 h. After incubation, the tubes were centrifuged at 5000 rpm (Centrifuge 5424, Eppendorf AG, Hamburg, Germany) for 5 min. Then, PBS medium was discarded and 2 mL of fresh PBS (pH 7.4) was added to remove all the unattached gentamicin sulfate molecules [25]. Two milliliters of isopropyl alcohol (Sigma-Aldrich, Steinheim, Germany) and another 2 mL of phthaldialdehyde reagent were added to the fresh PBS. The gentamicin sulfate complex concentration was detected by an UV-vis spectrophotometer (Hitachi, U-2000, Hachioji City, Tokyo, Japan) at 332 nm [30]. The gentamicin standards were prepared by dissolving appropriate amounts of gentamicin (10, 100 µg/mL and 1 mg/mL) in PBS. The release rate of gentamicin from the SiO_2_-gentamicin nanohybrids was detected throughout 5 days. Statistical analysis was conducted using the statistical package of Microsoft Excel 2013 (Office Professional Plus 2013, Impressa Systems, Santa Rosa, CA, USA). Each mean value was calculated from 3 consecutive measurements. The standard deviations are represented by the ± values. Confidence interval of mean values was detected as 95%.

Various mathematical models were applied to analyze the pharmacokinetic release profile of the gentamicin from the SiO_2_-gentamicin nanohybrids. These models are: (1) zero-order model; (2) first-order model; (3) Higuchi square root of time model; and (4) Korsmeyer-Peppas model as shown by Equations (1)–(4), respectively:

(1) $$F=K^{*}t$$

(2) $$F=1-e^{-k^{*}t}$$

(3) $$F=K^{*}t^{1/2}$$

(4) $$F=K^{*}t^{n}$$

In the equations, F is the drug release fraction at time t ($F=\frac{\%Q_{t}}{\%Q_{\infty }}$ in which $\%Q_{t}$ is the drug-released percentage at time t and $\%Q_{\infty }$ is the total drug-released percentage) and $K^{*}$ represents the release rate constant. The exponent (0.5, 1 or *n*) is related to the diffusional processes that could describe the different mechanisms of gentamicin release. The value of the exponent changes according to the release mechanism. In case of Fickian diffusion n≤0.5, for anomalous transport 0.5<n<1 and for zero-order release n=1 [20,22]. Regression analysis was used to detect which model corresponds to our *in vitro* release data.

### 3.3. Comparison of the Antimicrobial Action of the SiO_2_-Gentamicin Nanohybrids Versus Native SiO_2_ NPs and Free Gentamicin

#### 3.3.1. Agar Diffusion Assay

Antimicrobial susceptibility testing is most commonly performed using the agar diffusion method, as recommended by the Clinical and Laboratory Standards Institute (CLSI) [31]. The sensitivity of the bacteria to the tested materials was determined by measuring the inhibition zones produced by diffusion of the antibacterial agents from the wells into the surrounding medium. The results were interpreted according to the procedure presented by Winn *et al.* [32] and Quinn *et al.* [33].

Three different bacterial species from the culture collection of the Department of Biotechnology and Chemical Technology, Aalto University were used as representatives of gram-positive and gram-negative bacteria; namely, *B. subtilis* (TKK 10151), *P. fluorescens* and *E. coli* (VTT E-94564) that were stored in −80 °C. The bacterial species were sub-cultured on Nutrient agar (Lab M Limited^®^, Heywood, UK). Colonies obtained from the agar plates after 24 h incubation were inoculated into Mueller Hinton broth (Lab M Limited^®^). The bacterial concentrations were detected by measuring the absorbance using an UV-vis spectrophotometer (Hitachi, U-2000, Tokyo, Japan) at 600 nm [34]. Since the optical density measures the viable and dead bacteria in suspensions and as the traditional criterion to differentiate viable from dead bacterial cells is the ability of viable cells to produce a colony [35], our suspensions were obtained from 24-h cultured plates and the cultured broth was adjusted to match 0.5 McFarland standard. A volume of 100 µL of each bacterial suspension was spread on the Muller Hinton agar plates and 20 µL of the different tested materials was dispensed in the wells of the plates. Incubation of the plates was executed for 24 h at 28 °C for *B. subtilis* and *P. fluorescens*; and at 37 °C for *E. coli*. The agar diffusion assays were performed in duplicate and the inhibition zone diameters (mm) are the average of the measurements. Standard deviations of the inhibition zones (mm) are represented by the ± values.

Sterilization of the powder materials took place through UV-irradiation using an UV Chamber (GS Gene Linker^®^, Bio-Rad, Hercules, CA, USA). Then, all the UV-irradiated samples were dispersed in sterile de-ionized water in concentrations of 1 mg/mL for the microbiological tests. The agar diffusion assays were performed at a starting concentration of 1 mg/mL of the tested materials to screen for the antimicrobial action of the SiO_2_-gentamicin nanohybrids in comparison with the native SiO_2_ NPs and the free gentamicin. Then, the concentrations of the tested materials were decreased to detect the MIC in the broth microdilution assay. The tested materials were sonicated (power 234 W, working frequency 47 kHz ± 6%, Bransonic^®^, 2210E-DTH, Derwood, MD, USA) for 30 min before the antimicrobial applications to obtain homogenous solutions.

#### 3.3.2. Broth Microdilution Assay

MIC is the minimal inhibitory concentration of antimicrobial agent that inhibits bacterial growth [13]. The standard microdilution method was used to determine the MIC of the SiO_2_-gentamicin nanohybrids, native SiO_2_ NPs and free gentamicin according to the CLSI [31]. The original concentrations of the tested bacterial suspensions were equivalent to 0.5 McFarland standard. Within 15 min of the inoculum preparation, tenfold serial dilutions were performed in Mueller Hinton broth to reach 1.5 to 3 × 10^5^ CFU/mL. For the tested materials, twofold serial dilutions were performed from 1 mg/mL to 0.98 µg/mL. A volume of 100 µL of the different tested materials was added from the concentrations of 1000, 250, 62.5, 15.63, 3.91 and 0.98 µg/mL into the wells of the microdilution plate. Then, 10 µL of the bacterial suspensions were inoculated into the wells. Mueller Hinton broth without any added antimicrobial material was used as a negative control and only bacterial suspensions were used as a positive control. The results of MIC were read after 24 h of incubation.

Parallel antimicrobial tests (Agar diffusion assay and MIC) were performed for the free gentamicin sulfate sterilized by filtration through 25 mm syringe filter w/0.2 µm cellulose acetate membrane (VWR International, Wallkill, NY, USA). These tests were done to detect the possible deteriorating effect of the UV-irradiation on the loaded-gentamicin and whether it can be used as a sterilization option for antibiotic-conjugated nanoparticles or not.

#### 3.3.3. TEM Interaction Studies of the SiO_2_-Gentamicin Nanohybrids, Native SiO_2_ NPs and Free Gentamicin with the Bacterial Cells

Microscopic interaction of the tested materials with the gram-positive bacterium, *B. subtilis* and the gram-negative bacterium, *P. fluorescens* was investigated by TEM. The study included four groups. The first group consisted of the native bacterial cells that acted as a control negative. The second, third and fourth groups were the bacterial cells treated with the native SiO_2_ NPs, SiO_2_-gentamicin nanohybrids and free gentamicin, respectively.

Bacterial cells from the 24 h-cultured agar plates were inoculated into Muller Hinton broth and incubated at 28 °C for 24 h at 200 rpm in an incubator shaker (Lab-Therm, Fennolab, Kühner, Basel, Switzerland). 10 µg/mL of the tested materials was added to the bacterial suspensions [36,37] and re-incubation for a further 24 h was performed. The incubated bacterial suspensions with the tested materials (5 mL) were centrifuged at 10,000 rpm for 5 min, washed and re-centrifuged [38]. The samples were fixed by adding 0.5 mL of 2.5% gluteraldehyde at room temperature and collection of cells was accomplished by centrifugation at 13,000 rpm for 3 min. The supernatant was discarded and another 0.5 mL of 2.5% glutaraldehyde was added to the bacterial cells, after which the pellets were kept at 4 °C overnight. Washing of glutaraldehyde was performed using sodium phosphate buffer. Then, 1% osmium tetroxide was used as a second fixative for 1 h at room temperature. Washing of osmium tetroxide was performed two times by sodium phosphate buffer. The dehydration process was accomplished by increasing ethanol concentrations (50%, 70%, 96% and 100%) and finally by acetone (100%). Infiltration of the samples was implemented in ascending series of Epon^®^:acetone mix, starting with 30% for 3 h, followed by 70% and finally two changes of 100% epon, 3 h for each. Polymerization of the samples was performed in 60 °C oven for 14 h. After polymerization, the epon blocks were cut into thin sections of 60 nm thicknesses using an ultramicrotome (Leica, EM Ultra Cut UC6ei, Leica Mikrosysteme GmbH, Vienna, Austria). The sections were placed on grids (Formvar carbon film on 300 mesh-Cu grids, Electron Microscopy Science, Hatfield, PA). The sections on the grids were stained with 0.5% uranyl acetate, followed by 3% lead citrate for TEM micrographs.

## 4. Conclusions

We have demonstrated that the gentamicin of the SiO_2_-gentamicin nanohybrids represents 11.7 wt % payload that permits the fast gentamicin release of 21.4% during the first 24 h and extended release of 43.9% over the five-day experiment. This fast release in the initial stage suggests that SiO_2_ NPs are promising carriers for antibiotics and may have possibilities in the treatment and prophylaxis of infections associated with orthopedic applications that require high initial concentration of antibiotic for treatment followed by extended release. Coupling the antimicrobial tests of the SiO_2_-gentamicin nanohybrids with the *in vitro* release experiments is essential to apply the nanohybrids with concentrations that would ideally release the therapeutic levels of the antibiotic. This could avoid the crucial problem of releasing sub-inhibitory concentrations of the antibiotic. The SiO_2_-gentamicin nanohybrids are more potent against the gram-positive *B. subtilis* than the gram-negative *P. fluorescens* and *E. coli* when tested as pure cultures on laboratory media. UV-irradiation is a convenient method for sterilization of the nanoparticles loaded with gentamicin with no adverse effect on the antimicrobial properties of the antibiotic.

This study strives to contribute to the research targeting the facile preparation of SiO_2_ NPs to be used as antibiotic carriers that can permit the extended antibiotic release for efficient antimicrobial administration in different orthopedic applications. The present data have shown the different sensitivities of bacteria to the released antibiotic; however, it is evident that more detailed studies on a wider range of bacteria, preferably isolated from patients with osteomyelitis and capable of biofilm formation should be studied. In order to study the full potential of the developed materials, further work on bacterial strains relevant for osteomyelitis, especially the gram-positive *Staphylococcus species* is called for. Moreover, co-culture and bacterial interactions with bone and bone implants would also be of interest. Furthermore, longer *in vitro* antibiotic release studies should be concurrently performed for each specific clinical application of the SiO_2_-gentamcin nanohybrids. Our future research is directed towards the *in vitro* and *in vivo* toxicological evaluations of the SiO_2_-gentamicin nanohybrids to ascertain their more extensive application potential.

## Figures and Tables

**Table 1:** Calculated release rate constants (K_s_) and correlation coefficients (*R*^2^) after fitting gentamicin release profile, expressed by various mathematical models.

Mathematical Models	K_s_	*R*^2^
Zero-order model	2.5167	0.9662
First-order model	1.1519	0.9655
Higuchi model	0.8583	0.9721
Korsmeyer–Peppas model	0.966	0. 9769

**Table 2:** MIC of the SiO_2_-gentamicin nanohybrids, native SiO_2_ NPs and free gentamicin.

Tested Materials	MIC (µg/mL)		
	*B. subtilis*	*P. fluorescens*	*E. coli*
Native SiO_2_ NPs	-	-	-
SiO_2_-gentamicin nanohybrids	250 *	250 *	250 *
Free gentamicin	0.98	3.91 to 15.63 ^¶^	3.91 to 15.63 ^¶^

Notes: (-) indicates that the tested material did not inhibit the bacterial growth. (*) represents that this concentration of the SiO_2_-gentamicin nanohybrids released 6.26 µg/mL gentamicin that inhibited the bacterial growth. (^¶^) indicates that the MIC of the free gentamicin is within this range (3.91 to 15.63) as the tested concentrations were widely separated.

## Supplementary Materials

The following are available online at www.mdpi.com/1996-1944/9/3/170/s1. Figure S1: X-ray diffraction (XRD) patterns of the native SiO_2_ NPs and SiO_2_-gentamicin nanohybrids, Figure S2: Antimicrobial activity of the: (A) native SiO_2_ NPs; and (B) SiO_2_-gentamicin nanohybrids *versus* free gentamicin against: (1) *B. subtilis*; (2) *P. fluorescens*; and (3) *E. coli*.
