# Supplementary material for: Silica-Gentamicin Nanohybrids: Synthesis and Antimicrobial Action
Source: Materials (Basel). 2016 Mar 5;9(3):170. doi: 10.3390/ma9030170 (PMC5456682; doi:10.3390/ma9030170)
Supplement: Supplementary file 1 [file materials-09-00170-s001.pdf]

Supplementary Materials: Silica-Gentamicin Nanohybrids: Synthesis and Antimicrobial Action

Dina Ahmed Mosselhy, Yanling Ge, Michael Gasik, Katrina Nordström, Olli Natri and Simo-Pekka Hannula

X-ray Diffraction (XRD) Analysis of the Native SiO_2 NPs and SiO_2-Gentamicin Nanohybrids

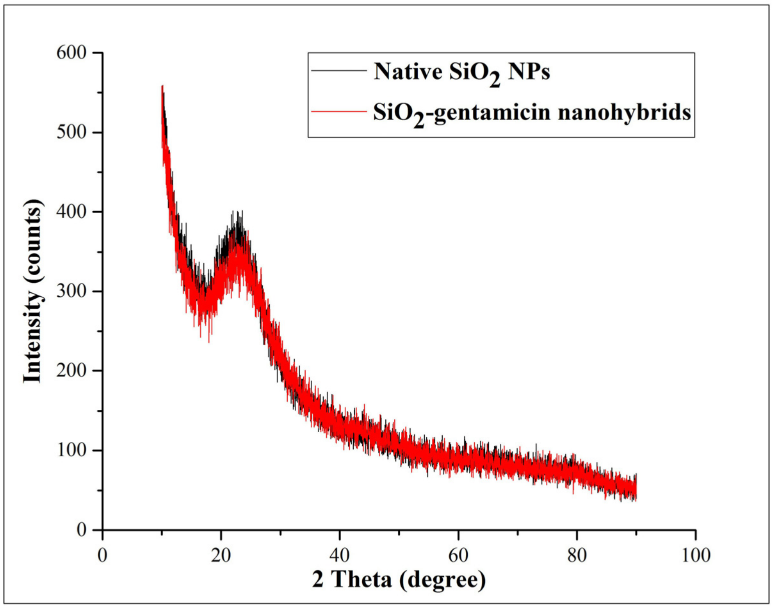

Figure S1. X-ray diffraction (XRD) patterns of the native SiO_2 NPs and SiO_2-gentamicin nanohybrids.

Agar Diffusion Assay of the SiO_2-Gentamicin Nanohybrids and Free Gentamicin

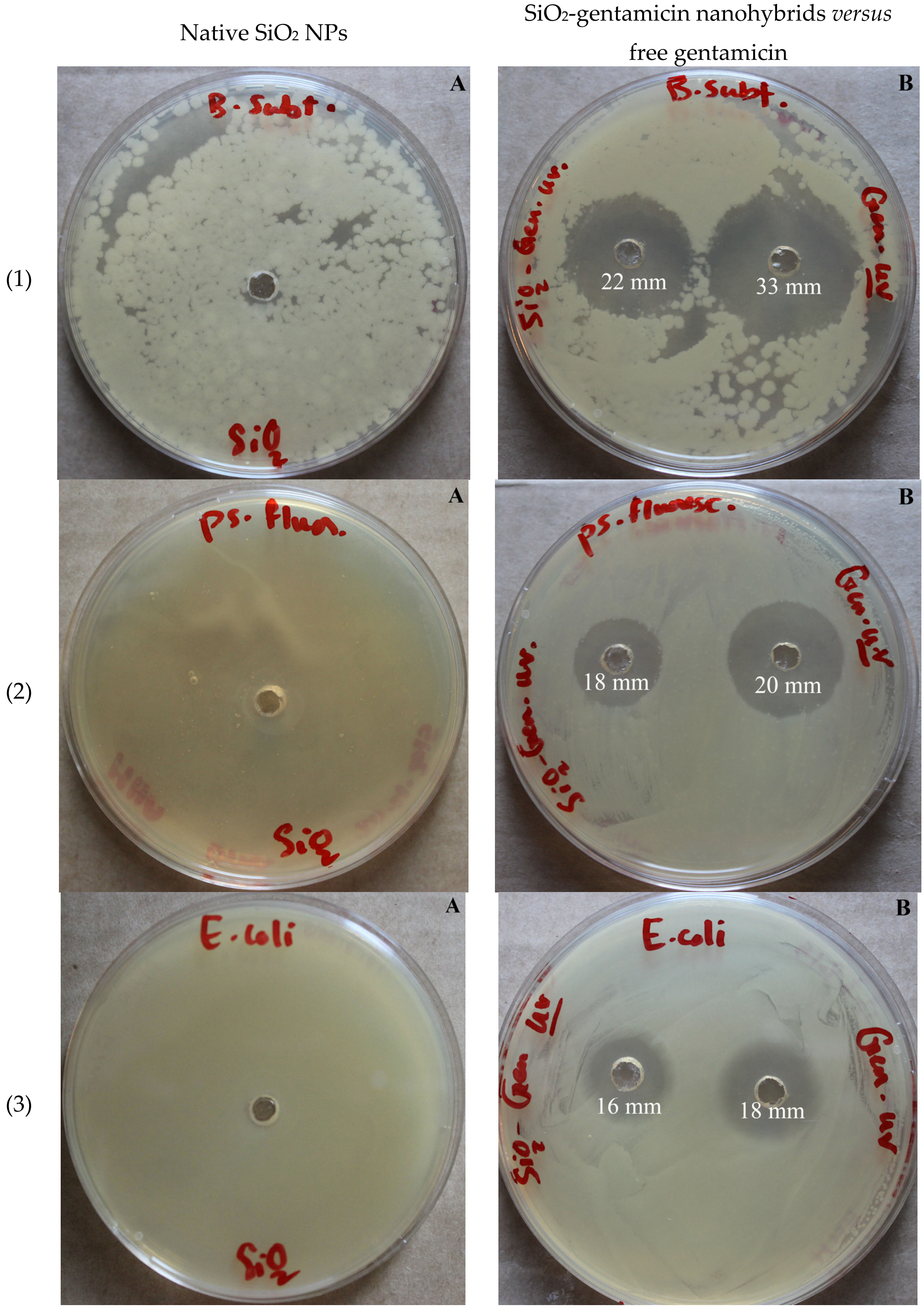

Figure S2. Antimicrobial activity of the: (**A**) native SiO_2 NPs; and (**B**) SiO_2-gentamicin nanohybrids *versus* free gentamicin against: (**1**) *B. subtilis*; (**2**) *P. fluorescens*; and (**3**) *E. coli*.
